# Video Consensus and Radical Prostatectomy: The Way to Chase the Future?

**DOI:** 10.3390/jpm13061013

**Published:** 2023-06-19

**Authors:** Francesco Esperto, Loris Cacciatore, Francesco Tedesco, Aldo Brassetti, Antonio Testa, Gianluigi Raso, Andrea Iannuzzi, Rocco Papalia, Roberto Mario Scarpa

**Affiliations:** 1 Department of Urology, Fondazione Policlinico Universitario Campus Bio-Medico di Roma, 00128 Rome, Italy; francesco.esperto@gmail.com (F.E.); francesco.tedesco@unicampus.it (F.T.); antonio.testa@unicampus.it (A.T.); gianluigi.raso@unicampus.it (G.R.); andrea.iannuzzi@unicampus.it (A.I.); rocco.papalia@policlinicocampus.it (R.P.);; 2 Department of Urology, IRCCS “Regina Elena” National Cancer Institute, 00128 Rome, Italy; aldo.brassetti@ifo.it

**Keywords:** prostate cancer, radical prostatectomy, written informed consent, video informed consent, awareness, surgery, patients

## Abstract

In the age of information, new platforms are consulted by patients to acquire consciousness about medical treatments. The aim of this study was to assess the level of understanding and feasibility of video consensus (VC) administration in patients scheduled for radical prostatectomy (RP), comparing it with standard informed consensus (SIC). According to the European Association of Urology Patient Information, we set up a video content for RP that was translated in Italian and implemented with information about possible perioperative and postoperative complications, days of hospitalization etc. From 2021 to 2022, all patients undergoing RP at our institution were prospectively included in this study. Patients received an SIC and after that, a VC about RP. After two consensuses were administered, patients received a preformed Likert 10 scale and STAI questionnaires. On the RP dataset, 276 patients were selected and 552 questionnaires for both SIC and VC were evaluated. Out of these, the median age was 62 years (IQR 60–65). Patients reported a higher overall satisfaction for VC (8.8/10) compared to the traditional informed consent (6.9/10). Therefore, VC may play a role in the future of surgery, improving the consciousness and satisfaction of patients and reducing preoperative anxiety.

## 1. Introduction

In the current technological era, especially following the COVID-19 pandemic where virtual strategies and “smart learning” activities are being used to continue education [1], the high availability of medical information through the web has further changed the health environment. Various health organizations have developed new technologies applied from diagnosis to treatment and even in follow-up [2,3]; patients increasingly consulted novel platforms to acquire their own consciousness about medical or surgical treatments, even if the information found is not always reliable.

In this setting, the Official Foundation of the American Urological Association (AUA) (https://www.urologyhealth.org; accessed on 28 April 2023) and the European Association of Urology (EAU) (https://patients.uroweb.org; accessed on 18 June 2023) have created educational contents based on the AUA Clinical Guidelines [4] and EAU Guidelines [5], respectively, in order to make available reliable information on various urological diseases. In the same way, the EAU Patient Information (EAU PI) working group delivers, always with the support of EAU guidelines, high quality video content about several surgical procedures in a language easy to understand for patients. Moreover, with the rise of advertising on social media, such as Instagram, Facebook, YouTube and TikTok [6], providing high quality clinical and surgical information could be the next challenge for medical community, in order to achieve greater knowledge and awareness by patients on their own health. Furthermore, TikTok is one of the fastest-growing social media apps in the urological landscape, even considering the paucity of quality information [7]. Xu et al. [8] evaluated the quality of information in the TikTok videos related to prostate cancer. The hashtag #prostatecancer was identified in 55 videos, which were subsequently reviewed and analyzed. Despite the high number of individual views (about 134,944), most of these videos (98.2%) were considered of low or bad quality. In more detail, in the TikTok videos that reported objective details, 41% contained a significant amount of poor-quality information, and thus misinformation. Additionally, 10.1% of the videos had an apparent commercial background. These reported results are worrying, since patients or family members receive distorted and fake information, which may wrongly influence the choice of health treatment.

Written informed consent is a cornerstone of modern health care. It is a conversation between surgeons and patients and/or relatives that allows them to make the best possible decision regarding a specific medical treatment. The lack of visual content in conventional standard informed consensus (SIC) may represent a barrier for patients for a full comprehension on the proposed procedure. Thus, the quest for the standardization of an all-inclusive consent with a detailed text and figures, safe graphics or video content, represents a new challenge in the medical scenario.

In this panorama, prostate cancer (PCa), the second most common cancer in men [9], is the utmost representation of technological innovation and communication through information sources, due to the undeniable improvement and employment of robotic platforms [10,11,12,13,14,15]. Additionally, PCa surgery has a strong impact on men’s global health, affecting social and sexual life, and often patients are more worried about potency and continence status outcomes rather than oncological success.

Therefore, in our study we attempted to assess the level of understanding and feasibility of VC administration in patients scheduled for radical prostatectomy (RP), comparing it with standard written informed consensus and their overall satisfaction and preoperative anxiety.

## 2. Materials and Methods

### 2.1. Study Population

The study was based on the administration of a video content and a satisfaction questionnaire in addition to SIC after the admission to the Urology department of our institution. An ethical board approval was not mandatory. All patients enrolled were requested to be involved, on a voluntary basis, in the study, signing a willing document of inclusion. A prospective analysis of the RP database was performed. From January 2021 to December 2022, the baseline, demographic clinical and perioperative data from 319 patients who underwent laparoscopic RP at our tertiary-referral center were collected. We selected patients with a clear PCa histology after prostate biopsy and with locally confined disease based on preoperative imaging (CT scan and bone scan). All surgeries were performed by two experienced surgeons (R.P.; G.P.F.). Lack of demographic and baseline data (*n* = 25), incomplete questionnaires (*n* = 17) and blind patients (*n* = 1) were the exclusion criteria.

### 2.2. Design of the Study

**Figure 1:**
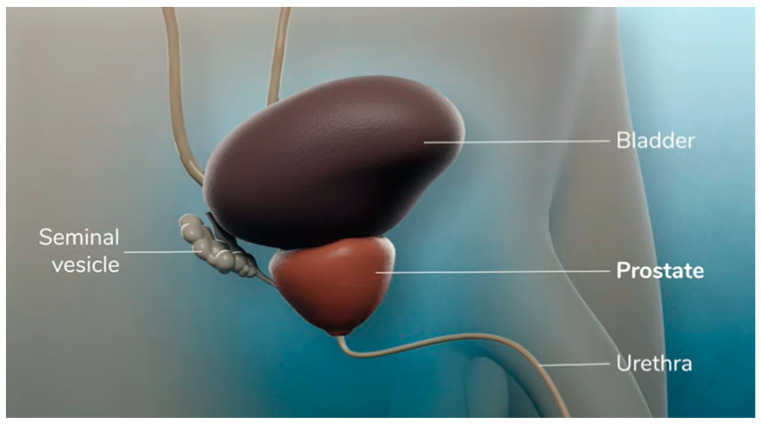
Freeze frame of the video that explains the anatomy of men in a simple way.

According to the EAU PI video content of RP, we created our own video, translated in the national language (Italian) from an Italian Urologist, a certified English speaker with a GMC license to practice and implemented with several information sections at the end of it, such as possible perioperative and postoperative complications, days of hospitalization and catheterization length. The video content lasted about 3 min and 30 s. Figure 1 shows a freeze frame of the video.

**Figure 2:**
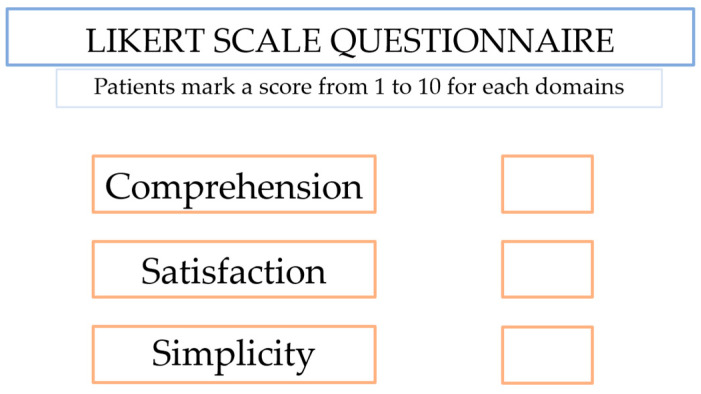
Likert scale questionnaire compiled by patients.

**Figure 3:**
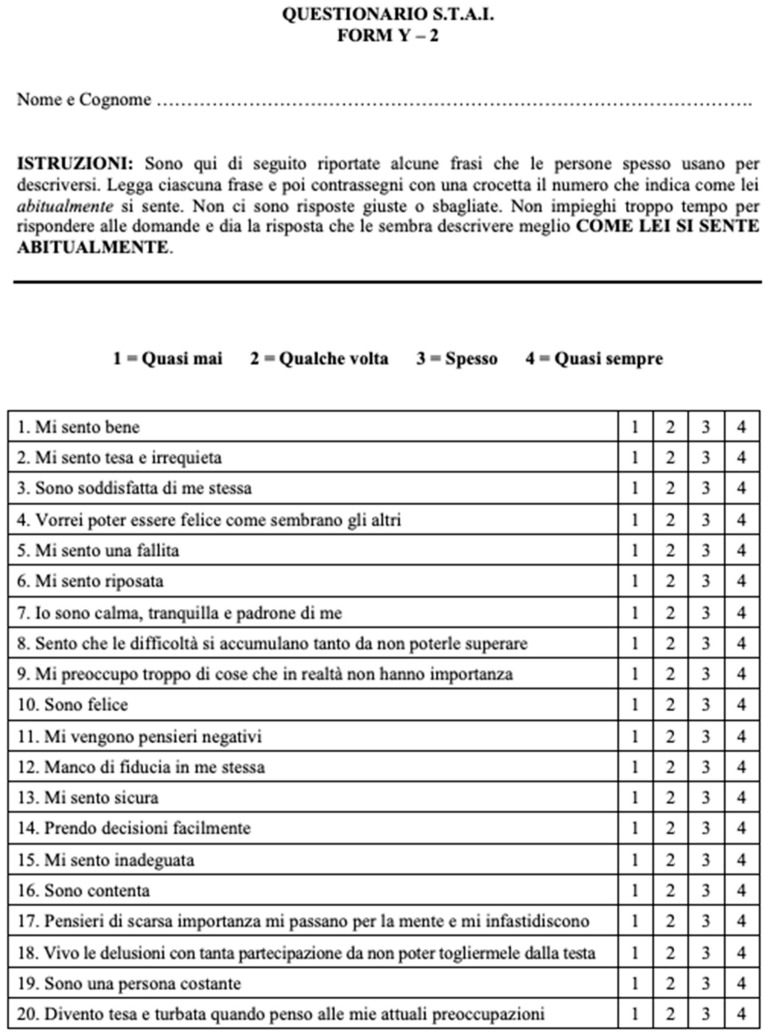
STAI questionnaire, Italian version, compiled by the patients: (Form Y2).

**Figure 4:**
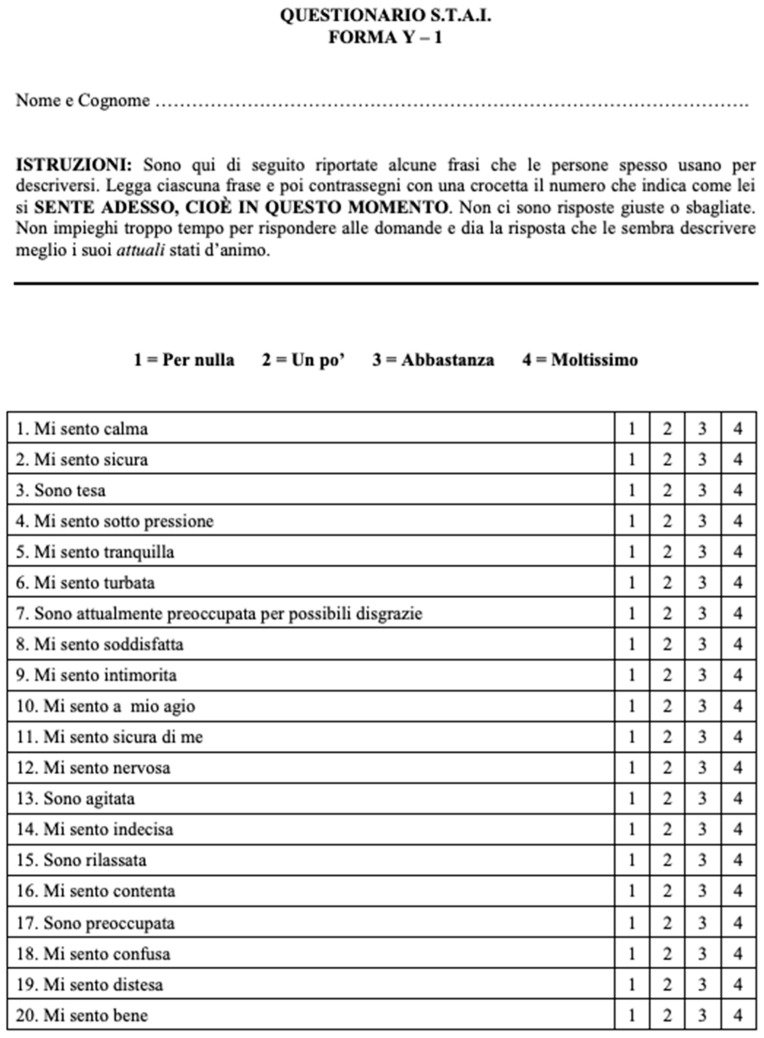
STAI questionnaire, Italian version, compiled by the patients: (Form Y1).

Our study was conceived as a four-step procedure:

- Firstly, patients received a print-based traditional consensus of RP administered from a physician in charge of explaining the surgical operation and possible complications.
- After the written traditional consensus, video content about RP with several added explanations was shown by a physician to the patient through an iPad.
- After written and video consent, patients filled in a preformed Likert 10 scale questionnaire (Figure 2) with a score from 1 to 10 for each consensus, to evaluate:
  - Comprehension
  - Satisfaction
  - Simplicity

- Finally, patients performed a State-Trait Anxiety Inventory (STAI) questionnaire (Spiel-berger, Gorsuch, Lushene, Vagg and Jacobs, 1983) [16], in the Italian version [17]. Figure 3 and Figure 4 show STAI questionnaire: patients performed the first part of STAI questionnaire (Y2) before the video consent and the second part of STAI questionnaire (Y1) after the explanation of the procedure through the video content.

Specifically, the STAI questionnaire is a self-assessment questionnaire that consists of a total of 40 questions, composed of 20 items assessing trait anxiety (Y2) (e.g., “I worry too much over something that really doesn’t matter”) and other 20 items assessing state anxiety (Y1) (e.g., “I am tense”, “I am worried”, “I feel calm”). In detail, state anxiety is related to how much the person feels anxious “right at that moment” and expresses a subjective feeling of tension and worrying, relational behavior of avoidance and an increase in the activity of the autonomic nervous system (increase in heart rate, galvanic response… etc.) relative to a stimulus situation, therefore transient and of variable intensity; while trait anxiety refers to how the subject usually feels, to a more enduring and stable condition of personality that characterizes the individual on an ongoing basis, regardless of a particular situation [18]. According to the 4-point Likert scale, patients evaluate on a scale of 1 to 4 (with 1 = for nothing and 4 = very much) how different statements fit their behaviors, with higher scores indicating more severe anxiety symptoms. In our series, the Y2 part was filled out by patients before viewing the video about RP; the Y1 was compiled after watching the video content.

### 2.3. Statistical Analysis

Continuous variables were presented as median and interquartile ranges (IQRs) and were compared using the Mann–Whitney U test, one-way based on their non-normal distribution. The descriptive and variance analysis was performed through the Statistical Package for Social Sciences (SPSS) software v.28.0 (IBM Corp, Armonk, NY, USA), with an alpha value of significance set at <0.05, comparing the different types of consent.

## 3. Results

Overall, 43 patients were excluded from the analysis. Consequently, a total of 276 patients were included in our study. We received and evaluated 552 questionnaires for both written and video-based consent and 276 STAI questionnaires. Concerning the clinical and operative features of patients, 91% (251 patients) were aged 50–70 years, 9% (25 patients) were over 70 years. The median patient age was 62 years (IQR 60–65). After the histological report, out of 276 patients, 53 patients (19.2%) had ISUP 1, 90 patients (32.6%) had ISUP 2, 86 patients (31.1%) had ISUP 3, 35 patients (12.7%) had ISUP 4 and 12 patients (4.4%) had ISUP 5.

We evaluated the mean score ± standard deviation (SD) for each domain of the Likert scale questionnaire between two informed consents. More in detail, the mean comprehension score was 6.87 ± (0.33) in the written consent group versus 8.47 ± (0.50) in the video consent group. The mean satisfaction score ± (SD) was 7.26 ± (0.44) in the paper-based consensus group versus 9.23 ± (1.31) in the VC group. The mean simplicity score ± (SD) was 6.56 ± (0.50) in the standard consensus group versus 8.74 ± (0.43) in the VC group (Table 1).

Additionally, the median and interquartile range were assessed for the same domains and were compared between two informed consents. Table 2 showed that the differences of each domain of the Likert scale questionnaire reached statistical significance between written and video consensus (*p* = 0.000). Afterwards, we also described a higher overall score reported by patients for video consent (8.8/10) compared to traditional informed consent (6.9/10).

Concerning the STAI questionnaire, the administration of the RP video content led to a decrease in preoperative patients’ anxiety, from a mean comprehension score ± (SD) of 66.4 ± (11.54) to a mean comprehension score ± (SD) of 52.1 ± (14.9), while the median and interquartile range were from 67 (IQR 63–78) to 53 (38–65), respectively. Table 3 showed the decreased level of anxiety of patients through the two forms of STAI questionnaire.

## 4. Discussion

The rise of patients’ awareness of PCa is related to a growing desire to know more about a disease that affects, potentially, not only overall survival, but also social and sexual life. Despite the invalidating sequelae on erectile function and urinary continence, RP remains the standard of care for the management of clinically significant PCa [19]. Indeed, prostate cancer constitutes a financial burden for both patients and the health care system [20,21,22,23]. A wide variety of information is available, but the role of the urologist remains hierarchical in the management of PCa and in improving patients’ awareness of their care pathway. Technological innovations represent a surplus for patients’ care with several techniques and strategies available for treatment, but at the same time they can often lead to more confusion and anxiety for the frequent conflicting information available in websites or social media, or for the presence of “fake news”, or for the lack of a critical knowledge background to evaluate the information received [7]. This has been widely shown on various social media platforms. Loeb et al. [24] assessed that only 54% of the videos on YouTube had medical terms and few reported some summaries or references, with a significant negative association between the scientific quality and viewer engagement (views/month *p* = 0.004; thumbs up/views *p* = 0.015). Finally, 77% of these reviewed videos attracted more public engagement (>6 million viewers), despite included latently misinformative and/or biased data within the video or comments section.

Social media analysis may help to understand how to reach our public more effectively and how use them in a positive manner [25], choosing the better platform for the different pathologies and creating specific contents describing details of diagnosis, treatment, or follow-up of diseases, in order to reduce general misinformation [26]. Therefore, in this technological scenario, a trust-based relationship between patients and urologists should be pursued to share good-quality information and decisions.

Written informed consent administration is a fundamental step before surgery, to let patients understand the scheduled procedure, possible complications and extinguish any doubts about it. Three substantial criteria are needed for an adequate informed consent: the patient must be knowledgeable, adequately informed and not obliged [27].

Moreover, before obtaining written informed consent for a surgical procedure, physicians need to let the patient know about the type of the surgery, the expected outcomes, material risks and adverse events, alternative surgical or non-surgical treatments, if available, and the consequences of them. As regards material risks, there are for each procedure specific risks and common risks for all surgeries, such as anesthesiologic troubles, blood loss, potential blood transfusions, infections etc. Otherwise, only in emergency scenarios can surgery be performed without informed consent, when the patient is not comprehensive and there is not available a substitute decision-maker [28].

Despite the doctors’ meticulousness during the consent administration, the lack of a full comprehension of the risk–benefit ratio by patients may represent an unsolved issue. Vikas et al. [29] assessed the level of patients’ information after the explanation of a traditional written informed consent. They described that no more than 75.14% of the participants were informed adequately regarding the type of surgery depending on age, educational level and annual income. Moreover, they reported that the totality of patients was well informed about their current clinical condition or pathology, while only 34% of patients were informed about risk and 26% about the alternative options of treatment.

In this background, we attempted to investigate the overall satisfaction and preoperative anxiety of patients undergoing RP at our institution, prior and after the administration and explanation of video content. Our evaluation showed interesting findings. First of all, the evaluation of the questionnaires on patients’ satisfaction allowed us to understand how much the patients’ awareness had significantly improved with the administration of video informed consensus compared to the SIC (*p* = 0.000). Our results are in line with those reported in the literature by previous studies concerning other fields of surgery, such as bariatric and trauma surgery [30,31,32]. This is probably due to the visualization of the surgical technique, with contextual explanation, that allows the patient to know step by step the proposed surgical procedure. Moreover, the use of a Likert scale questionnaire is an essential choice in the patients’ psychological assessment [33], as it allows us to evaluate three basic parameters of general satisfaction, in order to evaluate a new form of informed consent that explains more specifically the surgery. In the field of RP, countless videos on surgical techniques are available in the literature, such as several surgeries in motion [34]. However, this material is available to the urological community, but not to patients, who often look for information on websites or social media, enhancing anxiety and concerns about the procedure.

Additionally, concerning preoperative anxiety, our results of the STAI questionnaire showed a noteworthy median reduction in anxiety of 14 points after the administration of video informed consent. This may be probably due to the increased patients’ awareness of surgical procedure, but also to the active role of physicians during the video administration, explaining step by step the surgical procedure.

In the era of defensive medicine, a video consent model may reduce several medical–legal issues. Nowadays, several medical–legal issues are due to the lack of a scrupulous reading of written informed consent by patients, which leads to not understanding the procedure or any complications, providing a drawback between surgeons and patients. In this legal scenario, the Canadian Medical Protective Association reported that in a current period of 5 years, 65% of medical legal actions involving informed consent were towards surgical procedures and only 21% of these cases concluded in favor of the surgeon [35]. In this context, video-based informed consent could help to reduce this condition, based on the greater patients’ awareness of the procedure, especially of any intra- and post-operative complications. It would be interesting to consider whether in the future the standardized use of video consent could lead to a reduction in legal actions involving informed consent.

Unfortunately, our research study is not devoid of limitations. Firstly, our study included a single tertiary referral institution. A multicentric study would be ideal to confirm our findings. In our dataset we have not considered any neurological and/or psychiatric comorbidities that may lead the patient to have a state of anxiety independent of the surgery or to take medications for an anxiogenic condition. Finally, informed video consent could be considered time consuming and more expensive if compared to written informed consent. It requires the use of a laptop or of a tablet. Physicians and patients need time to visualize and discuss the video together.

Notwithstanding these limitations, to the best of our knowledge this is the first study conceived to assess the level of understanding and feasibility of video consent administration in patients scheduled for a urological surgical treatment. Furthermore, we considered in this study the largest series of video consent administration ever published, detailing preoperative satisfaction and anxiety.

## 5. Conclusions

Our results showed a higher patient satisfaction (mean score of 8.8 out of 10) of men for video consent compared to traditional informed consent (6.9/10). In the age of information, video consent represents a simple and comprehensive tool for patients, which improves their awareness and satisfaction and reduces the preoperative anxiety for the treatment chosen. Our study marks a new era of informative consensus through a shared scientific information supported by video content.

## Figures and Tables

**Table 1:** Mean score ± standard deviation of written and video informed consent related to the three domains of Likert scale questionnaire.

Domains of Likert Questionnaire	Written Informed Consent (Mean Score ± SD)	Video Informed Consent (Mean Score ± SD)
Comprehension	6.87 ± (0.33)	8.47 ± (0.50)
Satisfaction	7.26 ± (0.44)	9.23 ± (1.31)
Simplicity	6.56 ± (0.50)	8.74 ± (0.43)

**Table 2:** Median and interquartile range between the written and video informed consent related to the three domains of the Likert scale questionnaire.

Domains of Likert Questionnaire	Written Informed Consent (Median—IQR)	Video Informed Consent (Median—IQR)	*p* Value
Comprehension	7 (7–7)	8 (8—9)	0.000
Satisfaction	7 (7–7.5)	10 (7.75—10)	0.000
Simplicity	7 (6–7)	9 (8.25—9)	0.000

**Table 3:** Mean score ± standard deviation and median—interquartile range between written and video informed consent related to the STAI questionnaire.

Informed Consent	STAI Questionnaire (Mean Score ± SD)	STAI Questionnaire (Median ± IQR)
Before Video Informed Consent	66.4 ± (11.54)	67 (63–78)
After Video Informed Consent	52.1 ± (14.9)	53 (38–65)

## Data Availability

The data reported in this paper are available from the corresponding author upon request. Data is not publicly available due to privacy or ethical restrictions.

## Abbreviations

Prostate cancer (PCa); radical prostatectomy (RP); video consensus (VC); standard informed consensus (SIC); State-Trait Anxiety Inventory (STAI)
